# Prevalence and Correlates of HIV Infection among Street Boys in Kisumu, Kenya

**DOI:** 10.1371/journal.pone.0140005

**Published:** 2015-10-13

**Authors:** Ariella Goldblatt, Zachary Kwena, Maureen Lahiff, Kawango Agot, Alexandra Minnis, Ndola Prata, Jessica Lin, Elizabeth A. Bukusi, Colette L. Auerswald

**Affiliations:** 1 University of California, Berkeley–University of California at San Francisco Joint Medical Program, UC Berkeley School of Public Health, Berkeley, California, United States of America; 2 Center for Microbiology Research (CMR), Kenya Medical Research Institute, Nairobi, Kenya; 3 School of Public Health, University of California, Berkeley, California, United States of America; 4 Impact Research and Development Organization, Kisumu, Kenya; 5 Women's Global Health Imperative, RTI International, San Francisco, California, United States of America; 6 Bixby Center for Population, Health and Sustainability, School of Public Health, University of California, Berkeley, California, United States of America; British Columbia Centre for Excellence in HIV/AIDS, CANADA

## Abstract

**Introduction:**

Despite their perceived vulnerability to HIV, East African street youth have been neglected in HIV prevention research. We examined HIV seroprevalence and correlates of HIV infection in a sample of male street youth in Kisumu, Kenya.

**Methods:**

We enrolled a street-recruited sample of 13–21 year old street youth. Participants completed a survey followed by voluntary HIV counseling and testing. Survey items included demographics, homelessness history, survival activities, sexual behavior and substance use. We examined the relationship between predictor variables, markers of coercion and marginalization and HIV.

**Results:**

The sample included 296 males. Survival activities included garbage picking (55%), helping market vendors (55%), begging (17%), and working as porters (46%) or domestic workers (4%). Forty-nine percent of participants reported at least weekly use of alcohol and 32% marijuana. Forty-six percent of participants reported lifetime inhalation of glue and 8% fuel. Seventy-nine percent of participants reported lifetime vaginal sex, 6% reported lifetime insertive anal sex and 8% reported lifetime receptive anal sex. Twelve (4.1%; 95% CI: 2.3–7.0) participants tested positive for HIV. Of those, all had been on the street for at least one year and all had engaged in vaginal sex. Occupations placing youth at particular risk of coercion by adults, including helping market vendors (prevalence ratio (PR) = 8.8; 95% CI: 1.2–67.5) and working as domestic workers (PR = 4.6; 95% CI: 1.1–19.0), were associated with HIV infection. Both insertive anal sex (PR = 10.2; 95% CI: 3.6–29.4) and receptive anal sex (PR = 3.9; 95% CI: 1.1–13.4) were associated with HIV infection. Drug use, begging, and garbage picking were not associated with HIV infection.

**Conclusions:**

Although HIV prevalence in our sample of street youth is comparable to that of similarly-aged male youth in Nyanza Province, our findings highlight behavioral factors associated with HIV infection that offer opportunities for targeted prevention among street youth in East Africa.

## Introduction

The number of street children and youth (SCY) worldwide is unknown. Estimates vary widely [1]. SCY’s numbers are influenced by rapid population growth, poverty, urban migration, and the HIV/AIDS pandemic, all of which increase the risk of neglect of children in low-resource settings [2,3]. SCY are defined by UNICEF as either “of the street” or “on the street” [4]. “On the street” youth retain ties to their families and may return home to sleep at night. “Of the street” youth have limited ties to their families and live, sleep and work on the streets.

In sub-Saharan Africa (SSA), SCY are predominantly boys and young men and leave home at younger ages compared to street youth in developed countries [1,5–8]. Similarly, samples of SCY in Kenya have been mostly male. Ethnographic research has documented difficulties engaging girls, who are more likely to be absorbed into prostitution or other parts of the informal economy [3,9–13]. Prior to leaving home, SCY in SSA disproportionately experience poverty, family disruption, orphanhood, neglect and abuse, all of which increase their likelihood of leaving home and may increase their risk for HIV infection [3,5,6,8,14,15]. Of these experiences, orphanhood is particularly associated with increased risk of HIV infection in adolescents both in SSA and elsewhere [16–20]. A meta-analysis by Operario and colleagues, of which nine out of ten total articles were from SSA, found an HIV seroprevalence of 10.8% among orphaned adolescents compared with 5.9% among non-orphaned adolescents [17]. Kissin and colleagues found that street-involved youth who were both orphaned and homeless in St. Petersberg, Russia were three times more likely to be HIV positive than street-involved youth who were both sheltered and had living parents [21].

Once on the street SCY often join groups of street youth, who provide protection from harassment, and economic, emotional and social support for survival on the street [14,15,22–24]. Through their involvement in these groups, SCY are socialized into street-based survival activities, such as garbage picking, begging, petty theft and survival sex [1,5,14,15,22,25–27]. As many of these activities are stigmatizing or illegal, they lead to harassment or violence by the police and the public [25,28,29]. Furthermore, these activities place them at risk for accidental injury, malnutrition, and illness, including sexually transmitted infections (STIs) and HIV [1,3,26,27,30–32]. Kaime-Atterhög et al’s (2007) ethnographic findings from Nakuru, Kenya, two-hundred kilometers east of Kisumu, document distinctive social groups defined by street survival activities with the more marginalized “begging boys” contrasted with the “market boys” and “plastic bag sellers” [9,10]. They also note that boys might engage in sexual relationships with older female market vendors in exchange for food or fetching water [10]. In our own ethnographic work we also found increased stigma attributed to SCY who panhandled or engaged in garbage-picking as compared with other SCY [33]. We also noted that working for market vendors or as domestic workers rendered SCY dependent an informal economic relationship with an individual adult. These relationships rendered youth particularly vulnerable to coercion, relative to youth in informal economic relationships with multiple adults (for example, as garbage pickers or assistants to minibus drivers “matatu touts”).

Literature regarding HIV in SCY in SSA to date has documented their HIV-related attitudes, knowledge, and risk behaviors. Studies have found that street youth in SSA have lower levels of education and greater misconceptions about HIV transmission than their housed peers [7,34–36]. Additionally, many SCY do not perceive themselves to be at risk for HIV infection [36–39].

Furthermore, SCY engage in high rates of HIV-associated risky sexual behavior including early sexual debut, low rates of condom use, multiple partnerships, and survival sex (sex in exchange for money, food, shelter or protection) [7,10,26,32,37–42]. Rates of survival sex among samples of SCY in SSA have been reported to be between 21.7%-42.3% [26,37]. An example of survival sex in the ethnographic literature is the practice of kunyenga. Kunyenga described by Lockhart in Tanzania refers to survival sex among younger SCY who engage in anal sex with older youth in exchange for protection and admission into a group of SCY [10,28,43]. Kunyenga is not viewed as sex, but rather as a means of initiation or as a way to establish dominance. This practice may provide one survival-associated mode of HIV transmission among SCY.

Risky sexual behaviors are associated with substance use, with youth in the literature reporting both survival sex in order to obtain drugs, and unsafe sex while under the influence of substances [36,38,44]. Substance use, in turn, has been associated with HIV in SCY globally. Among homeless adolescents in the Americas, Eastern Europe and Asia, injection drug use (IDU) is a major mode of HIV transmission; however IDU is generally not as pervasive among SCY in developing nations as in developed nations [30,45,46]. Currently, IDU is rare among SCY in Western Kenya, though it is increasing rapidly in Mombasa and Nairobi [47]. SCY in SSA have high rates of alcohol, tobacco and marijuana use [12,44,46,48], as well as of inhalants, such as shoemaker’s glue and fuel, and of local stimulants, including khat and kola nut [1,30,36,44–46,48,49]. Sniffing glue is ubiquitous among SCY in East Africa, as well as in low-resource settings worldwide as it is inexpensive, omnipresent, and abates hunger and loneliness [15,22,27,50,51]. However, a link between HIV and substance use in SCY in SSA, and between HIV and inhalant use in particular, has not been documented.

Although research has documented the HIV-related attitudes, knowledge, and risk behaviors of SCY in SSA, data regarding their HIV seroprevalence are sparse. A recently published study by Winston et al measured HIV prevalence among street-connected adolescents in western Kenya and found an HIV seroprevalence of 14.8% among female participants in the study, although no male participants tested positive [52]. Other published studies of HIV seroprevalence among street youth in low-resource settings are primarily focused on Eastern Europe, Asia and the Americas [1,53–57], and are lacking in the context of a generalized pandemic. Data from the Ukraine and Nepal document HIV prevalence in SCY that is ten to thirty times higher than in the general population [55,56].

There are numerous barriers to the study of HIV seroprevalence and its determinants in SCY in SSA. First, SCY are a hard-to-reach population overlooked in household or school-based population surveys [14,27,56]. Second, SCY are highly stigmatized, leading to frequent criminalization rather than service-based approaches. Furthermore, the study of SCY requires careful consideration of ethical concerns, including the recruitment of out-of-home youth for HIV testing and the referral of HIV-positive youth for youth-appropriate care when such care is often hard to reach or simply not available [1,58,59]. Finally, current studies of street youth are often based on service-based convenience samples of youth who may have different risk profiles compared to street-based samples of youth [31,60].

Thus, despite their perceived vulnerability to HIV, East African street youth have been neglected in HIV prevention research. Accordingly, our study had two aims: 1) to describe the population of SCY in Kisumu, Western Kenya; and 2) to estimate their HIV prevalence and the correlates of HIV infection including participant’s vulnerability to coercion and marginalized status. In particular, we wanted to examine whether HIV prevalence increased with time on the street. If so, this would suggest a role for early intervention on the street to prevent new infections.

## Methods

### Study setting

We conducted our study in 2012 in Kisumu, the capital of Nyanza province, in western Kenya. Kisumu is Kenya's third largest city, located on the shores of Lake Victoria. With much of its economy based on subsistence farming, fishing and small-scale business, Nyanza is one of the poorest Kenyan provinces [16]. The overall HIV prevalence in Nyanza in 2013 was 15.1%, almost triple the national average of 5.6% [61]. In 2009, HIV seroprevalence among males 15 to 19 years old in Kisumu was 4%; among males aged 20 to 24 years old it was 13% [62].

### Preliminary studies

The current study is informed by a 2009 small mixed ethnographic and epidemiological pilot study [33,63] and by findings from a 2010 medical and service camp for SCY in Kisumu. The pilot study, for which we conducted HIV testing with 66 of-the-street males (median age of 14.9), demonstrated the feasibility and ethical acceptability of HIV testing in this population. Despite months of active street outreach to include “of-the-street” female SCY in pilot study activities, we only briefly located one, and were unable to enroll any. Our pilot ethnographic findings included a “social geography” of sites where SCY can be found and the street-based activities in which youth at each site are engaged. SCY use the Sports Grounds, a city park in Kisumu, to rest and sniff glue. The bus station, Kibuye market, Fanana Hotel, the Lakeside and near Barclays’ Bank are sites where youth engage in activities such as begging, garbage picking or assisting vendors. Common sleeping places for SCY are the bus station, and shopping centers including United Mall and Swan Center. Furthermore, our qualitative findings suggested that infection-associated risk behaviors among SCY may vary depending on whether they were engaging in more marginalized survival activities (i.e., activities associated with SCY status such as panhandling or garbage picking) or they were dependent on potentially exploitative economic relationships with adults (such as helping vendors in the market or household help) [33]. Findings from the pilot study, including the mapping and hypotheses, informed the design of the current study. The questionnaire and compensation used in the pilot study were adopted for use in the current study.

### Design and sampling

Our sample size calculation was limited by the absence of data regarding the HIV prevalence of SCY in East Africa. However, based on the prevalence of HIV among housed youth of similar ages in Kisumu and the overall prevalence of HIV in the province, we calculated the sample size that would allow for a 95% confidence interval for a range of HIV prevalence between 2%-15%, assuming the normal approximation could be used [61, 62, 64]. A sample of 300 was felt to give us ample power to estimate seroprevalence, with the caveat that a mobilization-based cross-sectional sample is not a random sample of youth.

The study sample was stratified by age category with goals of 75 participants, 100 participants and 125 participants for the 13–15, 16–18 and 19–21 year old age groups respectively. We set these quotas to ensure adequate representation of older youth, whom we hypothesized to be at greater risk for HIV. Older youth are ubiquitous on the street and in programs, however young street boys were far more likely to present themselves for recruitment in the pilot study, possibly because of their less lucrative options for survival. We describe below in the analysis section our adjustments to correct for our use of quota sampling.

### Eligibility and recruitment

Recruitment sites were identified by local outreach workers and informed by the pilot ethnography. Sites included the local bus station, market and fishing beach. Participants were recruited from street sites by study staff and outreach workers from local organizations serving SCY. Youth were also recruited by informal peer referral. Participants were eligible for the study if they were 13 to 21 years old and classified as “of the street” youth. We defined participants as “of the street” if they had spent no more than three nights in the previous week with their parents or guardian. Study staff were trained to identify SCY as ineligible if they were too distressed to answer questions or if they were under the influence of glue, alcohol or another substance. To prevent duplicate entry, participants were digitally fingerprinted [65] before completing a demographic and behavioral survey followed by voluntary counseling and testing (VCT) for HIV. Overall, nine potential recruits were found to be ineligible. Reasons for exclusion included duplicate enrollment (4 participants), intoxication (1), old age (1), “of the street” status (1), inconsistent answers to eligibility questions (1), and refusing fingerprinting (1). No participants were excluded due to emotional distress. Although gender was not an eligibility criterion, only one female was recruited and was consequently excluded from the analysis.

### Data collection

Participants completed an interviewer-administered, computer-assisted 72-item survey in Kiswahili, Dholuo or English. Surveys were completed in confidential booths in a meeting hall easily accessible to participants. Each survey took approximately fifteen minutes to administer and was conducted by one of four experienced Kenyan research assistants from the Kenya Medical Research Institute. After completing the survey, participants underwent rapid HIV testing using the national VCT algorithm [66]. Participants who tested positive received facilitated referrals to Tuungane Youth Clinic, which provides free HIV treatment and care. After data collection, HIV test results were matched to study participants’ survey data by unique identification number.

### Measures

The survey included measures of demographics, home life, street life, street survival activities, sexual behaviors, and drug use.

#### Demographic variables

included self-reported age; ethnicity; orphan status; and educational attainment, recoded as less than or equal to grade five versus greater than grade five. Grade five was chosen as the cut-off because all participants were of age at which they should have completed at least grade five and because it was the median education level in the sample.

#### Home life variables

characterized youth’s life before coming to the street, including parental occupations, food security prior to leaving home asked as “Before leaving home how many meals did you eat per day?” (three meals daily versus less than three meals daily), the presence of electricity at home, and reasons for leaving home.

#### Street life variables

included length of time on the street, type of shelter in the past week, and street survival activities (detailed below). Because an unexpectedly large percentage of youth reported having been on the street for over one year, time on the street was dichotomized as ≥ 1 year and < 1 year on the street. Examples of questions regarding time on the street and shelter in the past week included, “When was the last time you lived with your family every night?” with answers ranging from less than one week, one week to one month, one month to six months, six months to a year or longer than one year. Options for sleeping places included: with parents or another adult guardian, in a temporary shelter for children, outdoors (in a park, on the street, or another outdoor location where you were not sleeping for fun), in an abandoned building, in a vehicle, with a stranger or someone you did not know very well, or in jail or prison.

#### Survival activities

over the past three months were generated from our pilot study [33,63]. Examples of questions regarding survival activities included, “Which of the following ways in the last three months have you made money or cared for your needs?” These survival activities included begging, garbage-picking, helping market vendors, working as a domestic worker, working in beer halls, pickpocketing, acting as porters or “matatu touts” (assistants to informal bus drivers), and receiving money from family members or strangers.

#### Marginalization and vulnerability to coercion

To further explore our ethnographically-derived hypotheses regarding survival activities and HIV risk, we created qualitatively-informed variables for marginalization and vulnerability to coercion. Marginalization identified participants engaged in highly stigmatized survival behaviors associated with SCY, specifically garbage picking or begging. Vulnerability to coercion was defined as working as a domestic worker and/or for a market vendor.

#### Sexual behavior variables

included measures regarding vaginal sex, insertive anal sex and receptive anal sex. For each behavior, youth were asked whether they had ever engaged in the behavior, age at debut, whether they had used a condom at last intercourse, whether they had ever engaged in the behavior for survival, whether they had engaged in the behavior over the last three months, and, if so, with how many partners. Examples of questions regarding sexual behavior variables included: “Have you ever had vaginal sex?”, “How old were you the first time you had vaginal sex?”, “Have you had vaginal sex in the last three months?”, and “How many people have you had vaginal sex with in the past three months?”

#### Drug use variables

included frequency of alcohol, marijuana, tobacco and khat use in the prior three months (less than weekly/at least weekly) and lifetime inhalant use of glue or fuel (ever used/never used). We solicited lifetime inhalant use of glue or fuel because these are highly stigmatized substances closely associated with SCY.

### Ethical approval

The study received approval from the institutional review boards at the Kenya Medical Research Institute, the University of California at San Francisco, and the University of California, Berkeley. Study staff obtained written consent from participants using a form that was read in participants’ preferred language. Participants who were unable to sign their name made a mark in the presence of a witness. Kenya’s National HIV testing guidelines allow for minors at least 15 years old to consent for VCT and for minors under the age of 15 to provide their own consent if engaged in behaviors that put them at risk for HIV infection [66]. Participants under 15 years old completed a short quiz to assess their understanding of the consent form, for which they had to receive a passing score of 70% to be eligible for study participation. No potential participant was excluded due to a low score. Participants received a meal voucher from a local food vendor for their participation, a level of compensation that was not found to be coercive in the pilot study.

### Statistical analysis

Estimating the HIV seroprevalence of SCY in Kisumu was challenging given that several key pieces of information were not available a priori, in particular the actual size and composition of the population of SCY in Kisumu. This is often the case in marginalized or hidden populations, such as populations of injection drug users, sex workers, or of the homeless. We discuss here our approach to arriving at an estimate of seroprevalence in an imperfect world. Because no gold standard for the population size is available, we estimated the possible size of the population in two ways. The first was through a modified Delphi method whereby estimates of the size of a population are arrived at through surveys of key informants [67,68]. Our survey of providers in Kisumu yielded an estimate of 500 to 1000 SCY in Kisumu on a given day. One estimate from a 1997 document of the status of street children in Kisumu cited an estimate of 4,000 SCY [69]. That estimate predated the election violence of 2007–2008 and subsequent events which were felt by our community collaborators to have decreased the size of the street population in the period during which the study was conducted, though no formal data are available to confirm or refute this assumption. Thus, in the absence of the actual population size we corrected for our estimates using several possible population sizes ranging from 500 to 2000 [70–72]. We used the normal approximation along with a finite population correction factor to estimate confidence intervals for HIV seroprevalence over a range of estimates for the total population size of SCY in Kisumu [73]. The finite population correction factor was used because investigators and community collaborators were confident that we had sampled substantially more than five percent of the total population of SCY in Kisumu, the cutoff required for the use of a finite population correction.

As stated above, our use of quotas was motivated by our prior pilot experience in which younger SCY were far more likely to present for enrollment. This was an obstacle to being able to assess the relationship between seroprevalence and age or time on the street. However, the use of quotas introduced the need to employ weighting to correct for a possible sampling bias. In 2010, we collaborated with local service providers on a day-long camp offering medical and social services for 310 SCY (Street Children Medical Camp Frequencies, Kisumu Kenya, unpublished; 2010), during which basic data were collected including age, gender and chief reason for presentation to the camp. In the absence of a census of the number or age distribution of SCY in Kisumu, the age distribution from the medical camp was employed to estimate age distribution as the only contemporaneous data source regarding the age distribution of SCY available to us. In addition to calculating the gross and weighted overall HIV seroprevalence, we also calculated estimates by age bracket (13–15, 16–18 and 19–21 years of age). Age brackets were employed rather than point estimates by age for stability given the small numbers for each age. Out of 310 participants, only 4 females attended this camp, validating our unsuccessful experience trying to recruit females into our pilot study.

Confidence intervals were calculated using the Wilson Score Interval for small sample sizes [73]. We anticipated small cell sizes because of the expected low prevalence of HIV in the sample given the low rates of HIV among adolescent males in the general population [61].

In order to examine the relationship between predictor variables and HIV status, we assessed associations using Fisher’s exact tests and generalized linear models to generate prevalence ratios (PR) [74]. We considered Fisher’s exact tests results to be significant if they had a p-value < 0.05. We included both unadjusted and age-adjusted models. We were unable to calculate prevalence ratios adjusting for time on the street as all participants who tested positive for HIV had been on the street for one year or more. We addressed missing responses to our survey questions by including a “did not respond” category in our analyses to test whether participants with missing responses differed from other participants. We employed STATA SE/12.1 to perform our analyses.

## Results

### Demographic characteristics (Table 1)

We recruited 300 youth. The final analysis includes the socio-demographic characteristics of the 296 male participants for whom HIV test results were available. Survey results for four youth, including for one positive youth, could not be accurately linked, due to clerical error. These four youth were dropped from the final study sample because of the inability to link HIV test results, our primary outcome, with the independent variables in the survey. The final sample included 82 participants in the 13–15 year old age group (111% of our target quota), 113 participants in the 16–18 year old age group (101% of the target quote) and 101 participants in the 19–21 year old age group (81% of the target quota).

**Table 1:** Socio-demographic characteristics of the sample population stratified by length of time on the street.

	<1 year on the street, n (%)	≥ 1 year on the street, n (%)	Total* n (%)
**Ages** (n = 296)			
Median age			17
13–15	39 (50)	41 (19)	82 (28)
16–18	28 (36)	83 (39)	113 (38)
19–21	11 (14)	89 (42)	101 (34)
**Orphan status** (n = 296)			
Both parents living	28 (36)	47 (22)	75 (25)
Maternal orphan	13 (17)	36 (17)	49 (17)
Paternal orphan	14 (18)	46 (22)	61 (21)
Double orphan	19 (24)	66 (31)	85 (29)
Did not respond	4 (5)	18 (8)	26 (9)
**Educational Attainment** (n = 296)			
≤ Grade 5	33 (42)	115 (54)	149 (50)
> Grade 5	44 (56)	84 (39)	128 (43)
Did not respond	1 (1)	14 (7)	19 (6)
**Home Life**			
**Electricity at home** (n = 291)	15 (19)	33 (15)	48 (16)
**Three meals/day at home** (n = 290)	42 (54)	153 (72)	195 (67)
**Mother’s Occupation** (n = 284)			
Small scale business	17 (22)	84 (39)	101 (36)
Urban laborer	9 (12)	32 (15)	41 (14)
Peasant farmer/agricultural laborer	7 (9)	27 (13)	31 (11)
Housewife	7 (9)	8 (4)	16 (6)
Professional	6 (8)	7 (3)	13 (4)
Unknown	28 (36)	51 (24)	82 (28)
**Father’s Occupation** (n = 282)			
Urban laborer	20 (26)	50 (23)	70 (24)
Skilled Laborer	6 (8)	34 (16)	40 (14)
Peasant farmer/agricultural laborer	9 (12)	28 (13)	37 (13)
Small scale business	3 (4)	25 (12)	29 (10)
Professional	9 (12)	14 (7)	23 (8)
Unemployed	2 (3)	6 (3)	8 (3)
Unknown	25 (32)	49 (23)	75 (25)
Reasons For Leaving home* (n = 296)			
Family conflict	50 (64)	93 (44)	144 (49)
Death/illness of a parent	22 (28)	86 (40)	108 (36)
Poverty	10 (13)	33 (15)	43 (15)
Did not feel safe	7 (9)	18 (8)	46 (15)
Unable to attend school	14 (18)	21 (10)	35 (12)
Just felt like it	2 (3)	19 (9)	21 (7)
Too much responsibility	3 (4)	12 (6)	15 (5)
Abandoned	3 (4)	6 (3)	10 (3)
Peer pressure	0 (0)	3 (1)	3 (1)
Stigma	1 (1)	1 (00)	2 (1)
Witchcraft	0 (0)	2 (1)	2 (1)
**Time on the Street** (n = 296)			
≤1 week	9 (12)	0 (0)	9 (3)
1 week and ≤1 month	16 (21)	0 (0)	16 (6)
>1 month and ≤6 months	35 (45)	0 (0)	35 (12)
> 6 months and ≤ 1 year	18 (23)	0 (0)	18 (6)
≥ 1 year	0 (0)	213 (100)	213 (73)
Did not respond	-	-	5 (2)
Sleeping Places in the last 7 days**			
Outside	63 (81)	170 (80)	238 (80)
Abandoned building/vehicle	15 (19)	38 (18)	54 (18)
Rented house	5 (6)	22 (10)	27 (10)
Shelter	3 (4)	6 (3)	10 (3)
Stranger	1 (1)	7 (78)	9 (3)
With family/adult guardian	2 (3)	6 (3)	8 (3)
Remand center/prison	0 (0)	3 (1)	3 (1)
Street Survival Activities**			
Garbage picking	45 (58)	118 (55)	164 (55)
Helping market vendors	36 (46)	126 (59)	162 (55)
Porters/Matatu touts	29 (37)	106 (50)	136 (46)
Begging	16 (21)	30 (14)	48 (16)
Money from strangers	7 (9)	14 (7)	21 (7)
Domestic worker	5 (6)	7 (3)	12 (4)
Working in beer halls	4 (5)	8 (4)	12 (4)
Pickpocketing	1 (1)	7 (3)	8 (3)
Money from family	4 (5)	4 (2)	8 (3)

*Does not sum to total due to missing responses from “time on the street”.

**Participants could choose more than one option.

Table 1 presents both the total proportions of demographic variables, as well as proportions by time on the street. As described below, time on the street could not be included in our regression analyses, so stratified proportions are presented here instead.

As illustrated in Table 1, most of our sample of male SCY were orphans from poor backgrounds who had left home secondary to family conflict or the illness or death of a parent, who were sleeping outdoors and who were surviving primarily by garbage picking, helping matatu (minibus) drivers, or assisting market vendors. Seventy-three percent of participants (n = 213) had been on the street for longer than one year. The majority of participants were of Luo ethnicity, reflecting Kisumu's ethnic make-up.

### Risk factors for HIV (Table 2)

As for Tables 1 and 2 presents both the total proportions of risk behavior variables, as well as proportions by time on the street. Seventy-nine percent of participants (n = 233) had engaged in vaginal sex. Far fewer had engaged in either insertive (n = 19, 6%) or receptive (n = 23, 8%) anal sex. Condom use at last vaginal sex was 26% (n = 60) versus 11% (n = 2) at last insertive and 4% (n = 1) at last receptive anal intercourse. Overall, 7% of participants (n = 20) had ever engaged in survival sex. Six percent of participants (n = 15) who had engaged in vaginal sex reported that their most recent episode of vaginal sex was for survival, versus 21% (n = 4) of those who reported that their most recent episode of insertive anal sex and 22% (n = 5) of those who reported that their most recent episode of receptive anal sex was for survival. Only two participants (1%) reported engaging in both vaginal and anal survival sex. Weekly alcohol (144, 49%), and marijuana (94, 34%) use, as well as lifetime glue use (137, 49%) were common. No injection drug use was reported.

**Table 2:** Reported sexual behavior and substance use stratified by length of time on the street.

	<1 year on the street, n (%)	≥ 1 year on the street, n (%)	Total*, n (%)
Circumcised (n = 293)	62 (79)	156 (73)	219 (74)
Prior HIV test (n = 293)	48 (62)	147 (69)	196 (67)
Newly diagnosed HIV infection (n = 12)	0 (0)	10 (83)	10 (83)
**Substance Use**			
≥ weekly alcohol use (n = 286)	24 (32)	119 (57)	144 (50)
≥weekly marijuana use (n = 274)	17 (24)	77 (38)	95 (34)
≥weekly tobacco use (n = 67)	9 (100)	52 (91)	62 (93)
≥weekly khat use (n = 270)	9 (12)	25 (13)	34 (13)
Lifetime glue use (n = 280)	30 (40)	107 (52)	137 (49)
Lifetime fuel use (n = 261)	5 (7)	19 (10)	24 (9)
**Vaginal Sex**			
Lifetime vaginal sex (n = 293)	49 (63)	183 (86)	233 (79)
Median age at first vaginal sex**	13 (range: 7–16)	14 (range: 7–20)	14 (range: 7–20)
Median number of vaginal sex partners**	2 (range: 1–5)	2 (range: 1–15)	2 (range: 1–15)
Vaginal sex in the last 3 months** (n = 233)	19 (39)	94 (51)	114 (49)
Condom at last vaginal intercourse**(n = 233)	17 (35)	43 (23)	60 (26)
**Insertive Anal Sex**			
Lifetime insertive anal sex (n = 293)	3 (4)	16 (8)	19 (6)
Median age at first insertive anal sex†	16 (range: 3–16)	17 (range: 13–20)	17 (range: 13–20)
Median number of insertive anal sex partners†	3 (range: 2–5)	2 (range: 1–5)	2 (range: 1–5)
Insertive anal sex in the last 3 months† (n = 19)	3 (100)	9 (56)	12 (63)
Condom use at last insertive anal intercourse† (n = 19)	1 (33)	1 (6)	2 (11)
**Receptive Anal Sex**			
Lifetime receptive anal sex (n = 292)	7 (9)	16 (8)	23 (8)
Median age at first receptive anal sex‡	14 (range: 7–18)	13 (range: 10–18)	14 (range: 7–18)
Median number of receptive anal sex partners‡	1 (range: 1–3)	2 (range: 1–4)	3 (range: 1–4)
Receptive anal sex in the last 3 months‡ (n = 23)	5 (71)	6 (38)	11 (48)
Condom use at last receptive anal intercourse‡ (n = 23)	1 (14)	0 (0)	1 (4)
**Lifetime exchange sex** (n = 296)			
Never	69 (88)	203 (95)	277 (93)
Vaginal exchange sex**	7 (14)	5 (3)	12 (5)
Anal exchange sex^	1 (13)	4 (17)	5 (15)
Both vaginal and anal exchange sex^^	1 (14)	1 (5)	2 (17)

*Does not sum to total due to missing responses from “time on the street”,

**of those reporting vaginal sex,

^†^ of those reporting insertive anal sex,

^‡^ of those reporting receptive anal sex,

^**^**^ of those reporting both insertive and receptive anal sex,

^**^^**^ of those reporting vaginal sex and insertive and receptive anal sex.

### HIV prevalence (Fig 1)

Twelve participants or 4.1% (95% CI: 2.3–7.0%) tested positive for HIV. Seroprevalence estimates did not vary significantly by age group. The age-weighted prevalence was 2.8% (95% CI: 0.9–4.7%). Results from finite population calculations with estimated total population sizes of 500, 1000, and 2000 did not change our findings. Of the participants, 196 (67%) reported prior HIV testing. Ten of the positive participants had newly diagnosed HIV infections.

**Fig 1:**
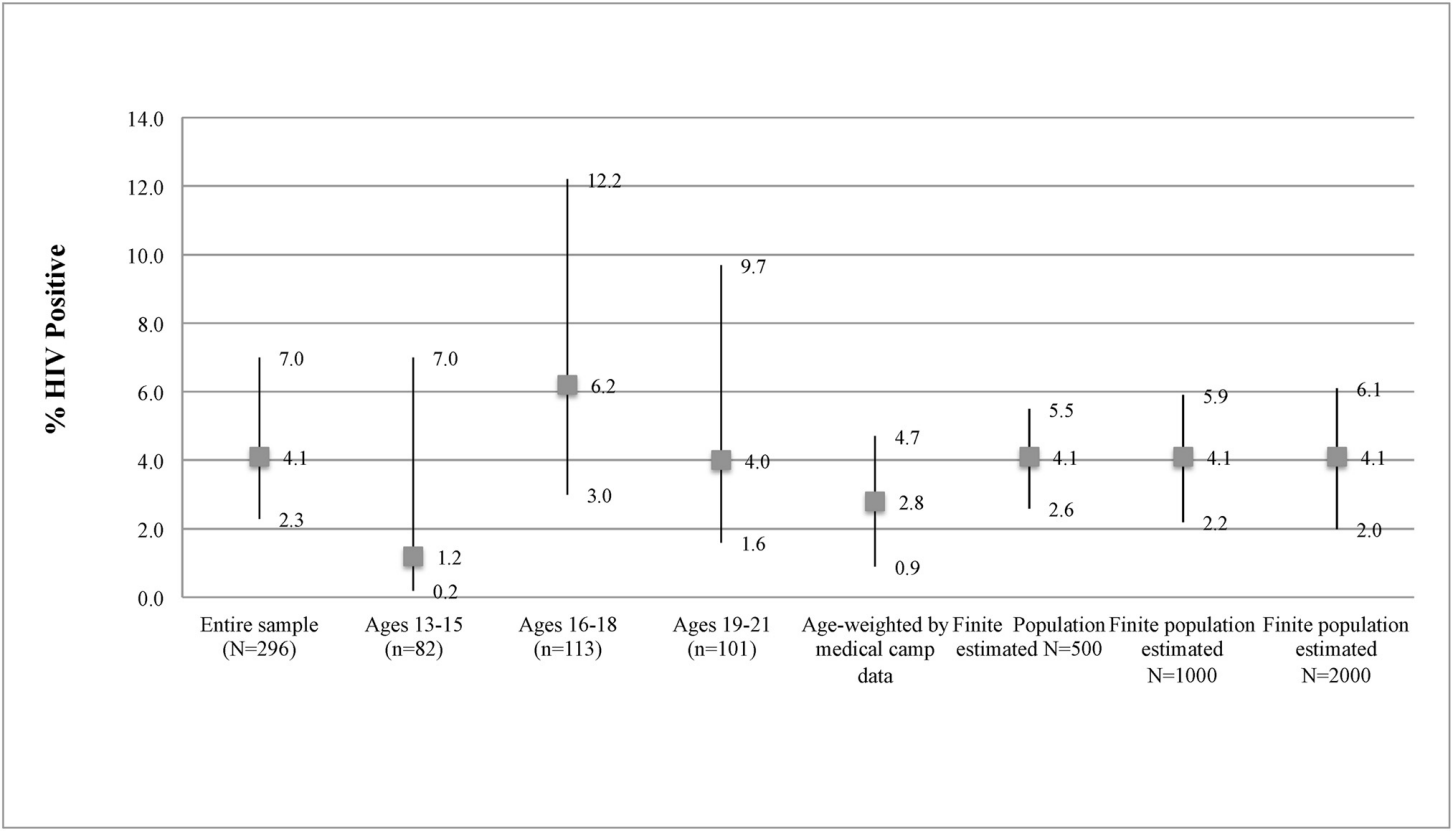
HIV Prevalence among Male SCY in Kisumu, Kenya.

### Socio-demographic correlates of HIV (Table 3)

No demographic or home life variables were associated with HIV infection, including orphan status. Although only marginally statistically significant (p = 0.077), all youth who tested positive had been on the street for at least one year.

**Table 3:** Socio-demographic Correlates of HIV Infection.

Variable	HIV + (N = 12)	HIV–(N = 284)	Fisher Exact Test P-value	Unadjusted PR (95% CI)	Age-adjusted PR (95% CI)
**Age**			0.206		
13–15	1	81		ref	
16–18	7	106		5.0 (0.6–40.5)	
19–21	4	97		3.2 (0.4–28.5)	
**Orphan status**			0.955		
Both parents living	3	72		ref	ref
Maternal orphan	3	46		1.5 (0.3–7.3)	1.5 (0.3–7.3)
Paternal orphan	2	59		0.8 (0.1–4.7)	0.8 (0.1–4.6)
Double orphan	3	82		0.9 (0.2–4.2)	0.8 (0.2–3.9)
Did not respond	1	25		1.0 (0.1–8.8)	0.0 (0.1–7.7)
**Educational Attainment**			0.778		
≤ grade 5	7	142		ref	-*
> grade 5	5	123		0.8 (0.3–2.5)	0.7 (0.2–2.3)
Did not respond	0	19		-*	-*
**Electricity at Home**			0.966		
No	10	233		ref	ref
Yes	2	46		1.0 (0.2–4.4)	1.0 (0.2–4.4)
Did not respond	0	5		-*	-*
**3 meals/day**			0.639		
No	5	90		ref	ref
Yes	7	188		0.7 (0.2–2.1)	0.6 (0.2–1.8)
Did not respond	0	6		-*	-*
**Time on the Street**			0.077	-*	-*
< 1 year	0	78			
≥ 1 year	12	201			
Did not respond	0	5			
**Street Survival Activities**					
**Begging**			0.264	-*	-*
No	12	232			
Yes	0	48			
Did not respond	0	4			
**Garbage picking**			0.473		
No	7	121		ref	ref
Yes	5	159		0.5 (0.2–1.7)	0.6 (0.2–1.8)
Did not respond	0	4		-*	-*
**Helping market vendors**			0.030		
No	1	129		ref	ref
Yes	11	151		8.8 (1.2–67.5)	7.6 (1.0–58.3)
Did not respond	0	4		-*	-*
**Porter/ Matatu Tout**			0.485		
No	8	148		ref	ref
Yes	4	132		0.6 (0.2–1.9)	0.5 (0.2–1.6)
Did not respond	0	4		-*	-*
**Domestic worker**			0.132		
No	10	270		ref	ref
Yes	2	10		4.6 (1.1–19.0)	5.0 (1.3–19.7)
Did not respond	0	4		-*	-*
**Money From Strangers**			0.391		
No	10	261		ref	ref
Yes	2	19		2.5 (0.6–11.0)	2.8 (0.7–11.7)
Did not respond	0	4		-*	-*
**Working in beer halls**			0.493		
No	11	269		ref	ref
Yes	1	11		2.1 (0.3–15.1)	2.0 (0.3–13.9)
Did not respond	0	4		-*	-*
**Money From Family**			0.768	-*	-*
No	12	272			
Yes	0	8			
Did not respond	0	4			
**Pickpocketing**			0.768	-*	-*
No	12	272			
Yes	0	8			
Did not respond	0	4			

* Not estimated due to zero cell.

The only survival activities that were associated with HIV infection were working as a domestic worker (PR = 4.6, 95% CI: 1.1–19.0; APR = 5.0, 95% CI: 1.3–19.7) and working as a market vendor (PR = 8.8, 95% CI: 1.2–67.5; APR = 7.6, 95% CI: 1.0–58.3).

### Risk behavior correlates of HIV (Table 4)

All youth who tested positive for HIV had engaged in vaginal sex, although this finding was not statistically significant. Having ever had insertive anal sex was associated with increased prevalence of HIV infection (PR = 10.2, 95% CI: 3.6–29.4; APR = 8.7, 95% CI: 3.0–25.2) as was having ever had receptive anal sex (PR = 3.9, 95% CI: 1.1–13.4; APR = 4.3, 95% CI 1.2–14.7). Ever having engaged in anal exchange sex was associated with increased prevalence of HIV infection (PR = 11.1, 95% CI: 3.2–38.1; APR = 10.3, 95% CI (3.2–32.9). Of note, no participants who reported ever engaging in vaginal exchange sex tested positive for HIV. Neither condom use nor substance use were associated with HIV infection.

**Table 4:** Risk Behavior Correlates of HIV Infection.

Variable	HIV + (N = 12)	HIV-(N = 284)	Fisher’s exact test P-value	Unadjusted PR (95% CI)	Age-adjusted PR (95% CI)
**≥ weekly alcohol use**			0.246		
No	4	138		ref	ref
Yes	8	136		2.0 (0.6–6.4)	1.8 (0.6–6.1)
Did not respond	0	10		-*	-*
**≥weekly marijuana use**			0.259		
No	5	174		ref	ref
Yes	6	89		2.3 (0.7–7.2)	1.9 (0.6–6.1)
Did not respond	1	21		1.6 (0.2–13.3)	1.9 (0.2–15.4)
**≥weekly tobacco use**			0.028		
No	1	4		ref	ref
Yes	5	57		0.4 (0.1–2.8)	0.6 (0.1–4.8)
Did not respond	6	223		0.1 (0.0–1.0)	0.2 (0.0–1.3)
**≥weekly khat use**			0.640		
No	10	226		ref	ref
Yes	2	32		1.3 (0.3–6.1)	1.4 (0.3–6.1)
Did not respond	0	26		-*	-*
**Lifetime glue use**			0.884		
No	7	136		ref	ref
Yes	5	132		0.7 (0.2–2.3)	0.7 (0.2–2.0)
Did not respond	0	16		-*	-*
**Lifetime fuel use**	10	262	0.447		
No	9	228		ref	ref
Yes	2	22		2.2 (0.5–9.6)	1.8 (0.4–7.9)
Did not respond	1	34		0.8 (0.1–5.8)	0.9 (0.1–6.7)
**Lifetime vaginal sex**			0.236	-*	-*
No	0	60			
Yes	12	221			
Did not respond	0	3			
**Lifetime insertive anal sex**			0.001		
No	7	267		ref	ref
Yes	5	14		10.2 (3.6–29.4)	8.7(3.0–25.2)
Did not respond	0	3		-*	-*
**Lifetime receptive anal sex**			0.089		
No	9	260		ref	ref
Yes	3	20		3.9 (1.1–13.4)	4.3 (1.2–14.7)
Did not respond	0	4		-*	-*
**Lifetime exchange sex**			0.041		
Never	10	267		ref	ref
Vaginal exchange sex	0	12		-*	-*
Anal exchange sex	2	3		11.1 (3.2–38.1)	10.3 (3.2–32.9)
Both	0	2		-*	-*

*Not estimated due to zero cells

### Correlation of vulnerability to coercion and of marginalization with HIV infection and HIV risk behaviors (Table 5)

As stated above, vulnerability to coercion was defined as working as a domestic worker and/or for a market vendor. Vulnerability to coercion was associated with ever using glue (PR = 1.3; 95% CI: 1.0–1.7, APR = 1.3; 95% CI: 1.0–1.7) and of having ever engaged in insertive anal sex (PR = 3.8; 95% CI: 1.1–12.9, APR = 3.4; 95% CI: 1.0–11.4). Additionally, every participant who reported ever having engaged in receptive anal sex also engaged in survival activities associated with coercion (p = 0.001). Vulnerability to coercion was significantly associated with HIV infection (PR = 7.9; 95% CI: 1.0–60.6, APR = 6.8; 95% CI: 0.9–51.4). Vulnerability to coercion was not significantly associated with ever having engaged in vaginal sex.

**Table 5:** Correlation of Vulnerability to Coercion and of Marginalization with HIV Infection and HIV Risk Behaviors.

	**HIV Positive**				
	No (n)	Yes (n)	P-value †	Unadjusted PR (95% CI)	Age-adjusted PR (95% CI)
**Coercion**			0.017		
No	121	1		ref	Ref
Yes	159	11		7.9 (1.0–60.6)	6.8 (0.9–51.4)
**Marginalization**			0.230		
No	109	7		ref	Ref
Yes	171	5		0.5 (0.2–1.5)	0.5 (0.2–1.6)
	**Lifetime Vaginal Sex**				
	No (n)	Yes (n)	P-value †	Unadjusted PR (95% CI)	Age-adjusted PR (95% CI)
**Coercion**			0.186		
No	30	92		ref	Ref
Yes	30	140		1.1 (1.0–1.2)	1.1 (0.9–1.2)
**Marginalization**			0.103		
No	18	98		ref	Ref
Yes	42	134		0.9 (0.8–1.0)	0.9 (0.8–1.0)
	**Lifetime Insertive Anal Sex**				
	No (n)	Yes (n)	P-value †	Unadjusted PR (95% CI)	Age-adjusted PR (95% CI)
**Coercion**			0.017		
No	119	3		ref	Ref
Yes	154	16		3.8 (1.1–12.9)	3.4 (1.0–11.4)
**Marginalization**			1.000		
No	109	7		ref	Ref
Yes	164	12		1.1 (0.5–2.8)	1.2 (0.5–3.0)
	**Lifetime Receptive Anal Sex**				
	No (n)	Yes (n)	P-value †	Unadjusted PR (95% CI)	Age-adjusted PR (95% CI)
**Coercion**			0.001		
No	122	0		ref	Ref
Yes	146	23		-*	-*
**Marginalization**			1.000		
No	107	9		ref	Ref
Yes	161	14		1.0 (0.5–2.3)	1.0 (0.4–2.1)
	**≥ Weekly Alcohol Use**				
	No (n)	Yes (n)	P-value †	Unadjusted PR (95% CI)	Age-adjusted PR (95% CI)
**Coercion**			0.720		
No	61	59		ref	Ref
Yes	80	85		1.0 (0.8–1.3)	1.0 (0.8–1.3)
**Marginalization**			0.276		
No	51	82		ref	Ref
Yes	90	51		0.9 (0.7–1.1)	0.9 (0.7–1.2)
	**≥Weekly Marijuana Use**				
	No (n)	Yes (n)	P-value †	Unadjusted PR (95% CI)	Age-adjusted PR (95% CI)
**Coercion**			0.607		
No	72	42		ref	Ref
Yes	106	53		0.9 (0.7–1.3)	0.8 (0.6–1.1)
**Marginalization**			0.795		
No	69	39		ref	Ref
Yes	109	56		0.9 (0.7–1.3)	1.0 (0.7–1.4)
	**Lifetime Glue Use**				
	No (n)	Yes (n)	P-value †	Unadjusted PR (95% CI)	Age-adjusted PR (95% CI)
**Coercion**			0.039		
No	68	48		ref	Ref
Yes	74	89		1.3 (1.0–1.7)	1.3 (1.0–1.7)
**Marginalization**			0.002		
No	68	41		ref	Ref
Yes	74	96		1.5 (1.1–2.0)	1.5 (1.2–2.0)
	**Lifetime Fuel Use**				
	No (n)	Yes (n)	P-value †	Unadjusted PR (95% CI)	Age-adjusted PR (95% CI)
**Coercion**			0.670		
No	101	9		ref	Ref
Yes	135	15		1.2 (0.6–2.7)	1.0 (0.5–2.3)
**Marginalization**			0.662		
No	95	9		ref	Ref
Yes	141	16		1.3 (0.6–3.0)	1.4 (0.6–3.1)

Does not add up to N = 296 because participants could choose more than one answer

*Not estimated due to zero cell.

^†^ Fisher’s Exact Test

Marginalization was defined as engaging in garbage picking or begging. Marginalization was associated with increased prevalence of ever having used glue (PR = 1.5; 95% CI: 1.0–1.7, APR = 1.3; 95% CI: 1.0–1.7). Although just approaching significance in the unadjusted model, marginalization was associated with a reduced prevalence of ever having had vaginal sex (PR = 0.9; 95% CI: 0.8–1.0, APR = 0.9, 95% CI: 0.8–1.0). Marginalization was not associated with HIV infection.

## Discussion

The goals of this study were to describe the population of SCY in Kisumu and to estimate their HIV prevalence and the correlates of HIV infection. Though the rate of HIV among SCY overall was not higher than the rates reported among male adolescents in Kisumu, our results suggest that a subgroup of SCY are at particularly high risk for HIV [75,76]. These high-risk youth exhibited a constellation of factors including surviving through activities that rendered them vulnerable to coercion (such as working as a domestic worker or for market vendors), engagement in receptive and insertive anal sex, and glue use (through its association with coercion and not directly through an increased risk of HIV infection).

The finding that the HIV prevalence in our sample was similar to the HIV prevalence among housed males in the region contrasts with seroprevalence data from other parts of the world, where HIV prevalence is markedly higher among SCY relative to housed youth [21,53,55–57,77]. Some reasons for this may include the lack of IDU among SCY in our sample, and the fact that Kenya, unlike the other countries in which concentration of HIV among SCY has been documented, is experiencing a generalized HIV pandemic. Although the percentage of single and double orphans among SCY was greater than in the community as a whole, orphan status did not confer increased risk for HIV for SCY, unlike the study in Russia. It could be that although orphans are disproportionately represented on the street in Kisumu, once on the street the risks for SCY do not differ by orphan status.

Robbins et al found an association between time on the street and the likelihood of being HIV-positive among Ukrainian street youth [54]. Although length of time on the street in our sample was only marginally significantly associated with being HIV-positive, all participants who tested positive had been on the street for at least one year. This finding deserves further exploration as it suggests, but (because of the marginal significance and cross-sectional nature of the data) cannot prove the existence of a hypothetical window of opportunity to prevent seroconversion among SCY, primarily by providing early avenues to exiting the street. A study by Ayaya and Esama in Western Kenya found that youth in shelters are less likely to engage in HIV-associated risk behaviors. Thus, shelters may offer a protective environment for SCY already on the street [29,30].

Although overall the rates of survival sex in our sample were lower that those reported among other samples of SCY in SSA, both insertive and receptive anal sex were predictive of HIV infection. Multiple ethnographic studies describe non-consensual anal sex engaged in for survival among SCY. Our previous ethnographic research, as well as Kaime-Atterhög’s findings from Nakuru Kenya, Lockhart’s findings from Mwanza, Tanzania and Mandalazi’s findings from Malawi, in which participants describe engaging in anal sex in return for food and shelter, all suggest that anal sex among SCY is often nonconsensual [10,24,43,33]. Further studies would be necessary to confirm a link between these initiation and survival activities and HIV risk.

Potentially coercive survival activities, such as working for market vendors or as domestic workers, were associated with HIV infection. These survival activities may be associated with sexual exploitation including coercive anal sex. The relationship between these survival activities and HIV infection deserves further exploration. Intervening in these possibly exploitative relationships by providing alternative income sources or by engaging youth in age-appropriate activities such as education and vocational training may help prevent HIV infection.

While HIV infection was associated with coercion, there was no relationship between HIV and marginalization. It may be that street youth engaged in stigmatized behaviors associated with marginalization are so isolated from the general population that they are actually protected from HIV infection in the context of a generalized, primarily heterosexually-transmitted pandemic. This is hinted at by their decreased likelihood of engaging in vaginal sex relative to youth not reporting marginalized behaviors, but this a hypothesis that would require additional qualitative and quantitative exploration.

The rates of glue use in our sample, a drug rarely used among housed youth in Kisumu, were very high [1,45]. Glue use was associated with both marginalization and coercion. The association of glue with coercion and coercion’s relationship with anal sex was described in our previous ethnographic work [33]. In qualitative interviews, participants describe younger boys or boys new to the street being intoxicated by their assailants with glue or being given glue in exchange for sex with experienced street-based youth leaders or adult men in the community [33]. This behavior may contribute to transmission of HIV infection to and among street youth. However, more research is needed to elucidate this relationship. Either way, structural interventions preventing glue use, including policies restricting the sale of shoemaker’s glue to minors, would benefit the health of street youth in East Africa [44, 48, 78].

A key strength of this study is that it is one of the first to our knowledge to describe HIV seroprevalence and its determinants in a street-recruited sample of East African SCY. Our study also demonstrated a high level of acceptability of street-based HIV testing in this population and the feasibility of referring new positives to youth-friendly care. Our finding that ten out of twelve seropositive youth were not aware of their infection also suggests that investments in testing in this population may yield new infections and provide an opportunity for early referral to care and treatment. Finally, our sample size relative to the estimated population size of SCY increases our external validity in Kisumu.

Our sample population shares overlapping characteristics with Winston et al’s recent work from Eldoret, Kenya with several important distinctions [52]. Unlike Winston et al’s study, our sample was entirely male. This might be explained by the fact that female SCY are less likely to be found in street settings, where we conducted our recruitment [3,9–13]. Additionally, none of the male participants in Winston et al’s study were HIV seropositive. Our higher prevalence of HIV could be due to our entirely street-based recruitment, which may have resulted in greater representation of non-service connected male SCY. The higher prevalence could also be due to the higher median age of our sample (17 vs. 15). However, given our finding of a low overall prevalence, it is possible that our results do not significantly differ from the findings in Eldoret.

Our study has several important limitations that limit the generalizability of our results. First, because of the lack of a sampling frame of SCY, we had to estimate our seroprevalence from a convenience sample. Second, our sampling strategy may have led biases in our findings. Our quota-based sampling to ensure the inclusion of older SCY may have artificially increased our estimated prevalence, as it is well documented that HIV prevalence increases with age in the general population of males in Nyanza province [62]. However, a relationship between age and serostatus was not found in our population. Conversely, it is also possible that our use of medical camp data for weighting may have artificially decreased our estimated prevalence given that older, sicker and more marginalized youth would be less likely to engage in such services. Nevertheless, the medical camp data were the closest approximation we had of a true age distribution for the population of SCY in Kisumu. Third, the low prevalence of HIV in this sample limited our power to conduct further sub-analyses. Fourth, our cross-sectional design limited our ability to elucidate causality. Finally, interviewer-administered survey questions are susceptible to social desirability bias, especially sensitive questions regarding stigmatized sexual behaviors and drug use.

## Conclusion

SCY are a vulnerable and neglected population in HIV prevention research in SSA. We found that the prevalence of HIV among street youth is comparable to the HIV prevalence among adolescent males overall in Kisumu. However, our findings regarding their vulnerabilities call for our attention and intervention. Several factors are associated with HIV infection, among them helping market vendors or working as domestic workers, engaging in insertive and receptive anal sex, and, though marginally, being on the street for over one year. Interventions that address SCYs’ susceptibility to economic and sexual exploitation (including providing them with alternative income-generating activities), interventions that remove them from the street through shelters or, when possible, reconnection to their communities of origin, and possibly interventions that limit their access to inhalants are necessary to reduce HIV risk in this population. We hope that our findings will encourage further research and the piloting of new, scalable interventions to promote the health and wellbeing of SCY and their re-integration into society.

## Abbreviations

IDU: injection drug use
KEMRI: Kenya Medical Research Institute
SCY: street children and youth
STIs: sexually transmitted infections
SSA: sub-Saharan Africa
VCT: voluntary counseling and testing
